# Vicarious Menstruation

**Published:** 1887-10

**Authors:** Duncan C. McCallum

**Affiliations:** M. D., M. R. C. S., Eng., of Montreal, Canada


### *VICARIOUS MENSTRUATION.*

By DUNCAN C. McCALLUM, M. D., M. R. C. S., Eng., of Montreal, Canada.

After a *résumé* of the literature of the subject and the diverse opinions of modern authorities, the reader cited four cases:

1st. Mrs. W., aged thirty-eight; six children; never nursed; good health. Two months after birth of child, had molimena and vomited blood. Treated by rest, ice and gallic acid. No unpleasant after-effects and no further hemorrhage for four weeks, when she again had molimena, followed by hæmatemesis. At next period, menses re-appeared, and have been normal since. Continued good health.

2d. Healthy woman; single. On the first day of a menstrual period, was exposed to cold, and menses stopped; next day, vomited blood; no vaginal discharge; regular since, and healthy.

3d. Patient aged thirty-three; healthy. First menstruation at fourteen years of age. Soon after had scarlatina, followed by amenorrhœa until eighteen. At twenty-three, menstruation became very scanty, and was accompanied by epistaxis for six periods, when it became regular again. Recently has again become scant, and is accompanied by the epistaxis as before.

4th. Healthy woman; pregnant three months. Six weeks before, had received a severe fright. Had a profuse hæmoptysis on two successive mornings, and three days later aborted; four weeks later, molimena and hæmoptysis, but since, normal menstruation. Chest perfectly sound; good health. In this case the ovum was killed six weeks before ovulation became established, and obstruction being offered to the usual flow, hemorrhage took place from the weakest point.

To constitute vicarious menstruation, there must be (*a*) absence of menstrual blood flow, (*b*) blood from some other organ than the uterus, and (*c*) no other assignable cause for the hemorrhage than the increased pre-menstrual blood-tension.

A hemorrhage under these conditions is truly supplementary and clearly vicarious.

Dr. Charles T. Parks, of Chicago, Ill., mentioned a case occurring in a single woman, twenty-three years of age; sick eighteen months. For four months, defecation had been at intervals of from one to seven weeks, and during this time only one ounce of urine had been passed daily. Severe fecal vomiting. No normal menstruation for two months, but at the time for the periods molimena and vomiting of pure blood. Patient was a physical and mental wreck. Exploratory abdominal incisions showed intestines filled with scybalæ. The ovaries, much enlarged, were removed. Urine at once increased to a pint daily, many scybalæ were passed, and general health rapidly improved. For two periods the patient had molimenæ and spat blood. Heart and lungs normal.

Dr. Opie, of Baltimore, Md., thought cases of vicarious menstruation rare and illy defined. He believed that when the menstrual flow was impeded, the vascular tension would seek relief at the weakest point.

Dr. Nelson, of Chicago, Ill., recalled a case where bleeding from rectal hemorrhoids occurred at menstrual periods, there being no uterine flow. The piles being cured and the cervix dilated, the menses appeared and gradually became normal.

Dr. Rodney Glissan, of Portland, Ore., in thirty-nine years, had seen three cases; in one, menstruation had been regular, but after a serious illness, hæmoptysis appeared every month, and no vaginal flow for a year, when menstruation again became regular, and the cough ceased. Now healthy.

